# Improving support in pregnancy after stillbirth or neonatal death: IMPs study

**DOI:** 10.1186/1471-2393-15-S1-A14

**Published:** 2015-04-15

**Authors:** Tracey A Mills

**Affiliations:** 1 School of Nursing, Midwifery and Social Work, The University of Manchester, UK

## 

The death of a baby before or shortly after birth is associated with profound long lasting grief for parents, similar to any child death.[1] The majority of women who suffer a stillbirth will embark on another pregnancy, with around 50% becoming pregnant within a year of the loss.[2] Subsequent pregnancies are characterised by elevated maternal anxiety and emotional vulnerability which often extends beyond the postnatal period, increasing the risk of adverse pregnancy outcomes and disrupted attachment, a potential cause of parenting and social difficulties in the longer term.[2-4] Recent evidence suggests the effects are not limited to mothers and that fathers also experience psychological distress during this period.[5]

A recent metasynthesis of the qualitative literature surrounding parents’ experiences of care highlighted the value of additional emotional and psychological support from healthcare providers in improving care in pregnancy after stillbirth or neonatal death.[6] However, there is a dearth of evidence to whether parents’ needs for emotional and psychological support are being met by current maternity services in the UK. This programme of research aimed to explore current UK practice and provision of support in pregnancy for parents following stillbirth or neonatal death. An action research approach was utilised with stakeholder involvement central to the design and conduct of the study.[7] Online surveys, including open and closed questions, provided an overview of current service provision in UK maternity units and women’s experiences. Qualitative phenomenological interviews provided an in-depth exploration of the lived experiences of women and health professionals. Data from 138 maternity units (≈60% total) demonstrated variable provision, emphasis on surveillance and monitoring with less attention to psychological and emotional support. A few units had developed innovative services/programmes targeted at this group, but lack of evaluation and dissemination was a barrier to sharing good practice. Analysis of the responses of 547 women, across all UK regions, demonstrated high levels of engagement and utilisation of maternity care. Many women reported positive experiences and recognised professionals who demonstrated empathy and compassion in providing high-quality care which often exceeded their expectations. However, a significant minority of women recounted poor experiences. Insensitive communication was often related to the attitudes and behaviours of individual professionals; however, organisational factors particularly a lack of continuity of care provider and service fragmentation common in standard UK model of ‘high-risk’ antenatal care were consistently and repeatedly associated with decreased satisfaction with care. Ongoing qualitative work will explore these issues in greater depth.

Interim findings of this programme raise the issue of equity in provision of appropriate and sensitive care for parents in subsequent pregnancies who utilise UK maternity services. Data suggests that many parents receive inadequate emotional and psychological support and therefore there is a need to improve the evidence base underpinning care. The findings of this study will directly inform the development of specific interventions to improve antenatal support and promote positive birthing experiences and the development of a clinical care pathway to improve the care of women and their families in pregnancy following perinatal loss.
